# Rural Women’s Experience of Living and Giving Birth in Relief Camps in Pakistan

**DOI:** 10.1371/currents.dis.7285361a16eefbeddacc8599f326a1dd

**Published:** 2017-01-31

**Authors:** Humaira Maheen, Elizabeth Hoban

**Affiliations:** School of Health and Social Development, Deakin University, Melbourne, Victoria, Australia; School of Health and Social Development, Deakin University, Melbourne, Victoria, Australia

## Abstract

Background: Women are more vulnerable than men in the same natural disaster setting. Preexisting gender inequality, socio-cultural community dynamics and poverty puts women at significant risk of mortality. Pregnant women are particularly vulnerable because of their limited or no access to prenatal and obstetric care during any disaster or humanitarian emergency setting.

Methods: In-depth interviews were conducted with 15 women who gave birth during the 2011 floods in Sindh Province, Pakistan. Thematic analysis explored women’s experiences of pregnancy and giving birth in natural disaster settings, the challenges they faced at this time and strategies they employed to cope with them.

Results: Women were not afforded any control over decisions about their health and safety during the floods. Decisions about the family’s relocation prior to and during the floods were made by male kin and women made no contribution to that decision making process. There were no skilled birth attendants, ambulances, birthing or breastfeeding stations and postnatal care for women in the relief camps. Women sought the assistance of the traditional birth attendants when they gave birth in unhygienic conditions in the camps.

Conclusion: The absence of skilled birth attendants and a clean physical space for childbirth put women and their newborn infants at risk of mortality. A clean physical space or birthing station with essential obstetric supplies managed by skilled birth attendants or community health workers can significantly reduce the risks of maternal morbidity and mortality in crisis situations.

## Introduction

Women are more vulnerable than men in the same natural disaster setting^1^^,^ 2. They are more likely to face physical or psychological trauma, compelled to live with strangers^3^^,^ 4 ^,^ 5, have decreased access to clean water and sanitation^3^, experience the loss of extended family members^6^^,^ 7, be victims of sexual and gender based violence^8^^,^ 9 and acquire communicable diseases^4^. Pregnant women face more challenges in any disaster setting because of their additional health care needs and limited access to health care facilities^10^^,^ 11. Following a natural disaster or conflict, adverse pregnancy outcomes such as miscarriages, premature delivery, still birth and postpartum hemorrhage are common as a result of women’s limited access to maternity care^12^.

The Minimum Initial Service Package (MISP) is a set of universal guidelines proposed by Women’s Commission for Refugee Women and Children in 2007, that address women’s reproductive health needs during the first phase of an emergency^13^. During humanitarian crisis, MISP recommends that a functional referral system be implemented which requires access to health facilities by means of adequate transportation to and from the relief camp^13^. The Inter-agency field Manual about reproductive health in refugee setting highlight the importance of training women and local TBAs to identify danger signs during pregnancy such as heavy bleeding, severe headache, blurry vision, swelling of face/hands, convulsions, prolonged labour, so that they can make timely and informed decision about seeking maternity care^13^.

For the population with mild to moderate level of malnutrition in a pre-crisis situation (10-15% of Global Acute Malnutrition), pregnant and lactating women should be provided food supplements along with the general rations to prevent them from becoming severely malnourished^14^. Branca stressed the importance of supporting lactating women to continue breastfeeding during disasters to prevent stunting and mortality amongst infants and neonates 15.

In the last decade Pakistan has experienced recurrent natural disasters (such as earthquake, floods, and drought) and since 2010 the natural disasters have become annual events^16^. The unprecedented flooding caused by monsoon rains in 2010 caused 1,985 deaths, damaged or destroyed 1.7 million houses and 1.4m acres (557,000 hectares) of agricultural land^17^^,^ 18 ^,^ 19 ^,^ 20. Approximately 485,000 pregnant women were affected by floods during 2010-13 and 50,000 required specialized obstetric care^20^. There are limited studies that have documented the challenges that women lived experiences during natural disasters or humanitarian emergency settings in Pakistan^16^ ^21^^,^ 22. Bukhari and Rizvi conducted a review of floods’ impact on women’s physical and emotional health by highlighting poor living conditions for women in relief camps across Pakistan^21^. Sadia et al., found that during floods, pregnant women’s utilization of private healthcare facilities were reduced due to poor road conditions, whereas utilization of rural health facilities (within 5km) and community health workers increased^16^. In both studies, authors did not present women’s birthing experiences in relief camp.

This study aimed to explore birthing experiences in relief camps of women who resides in disaster prone areas. The study findings can be used by the National Disaster Management Authorities and humanitarian agencies to make informed decisions about the provision of maternity care services in disaster or humanitarian emergency settings in rural Pakistan.

## Methodology

*Study site*

The study was conducted in five rural villages located along the bank of the Indus River of the Sindh Province of Pakistan. Due to their geographical location, the relative exposure to annual monsoon flooding is high in these villages. The human development and maternal health indicators of these villages were very low, which increases the residents’ vulnerability to respond to a natural disaster. The villages were geographically located close to a ‘district’ designated relief camp site which were established by the District Disaster Management Authorities (DDMA) during a crisis situation. These characteristics provided an ideal research site to explore maternity care challenges during the floods.

*Interview settings*

Purposive sampling is widely used in qualitative research to identify ‘information-rich’^23^ participants about a phenomenon of interest^24^. Women who gave birth during the floods between July 2011-September 2011 were selected as criterion of selection of participants for women interviews. There were 34 women who matched the criterion and were identified as a potential research participant during the preliminary visits to the community. Data saturation was used to calculate the sample size required for women’s interviews. I conducted a preliminary analysis with 10 interviews, and identify initial codes for analysis. I stop recruiting women after 15 interviews as the data was saturated.

In our initial research design, we decided to invite women for an individual interview. However, in the early stages of field research, and upon the wish of the research participants, we changed the interview method from an individual to a group interview. Group based discussions are recommended as a therapeutic way to collect information from persons who were victims of trauma^25^^,^ 26 ^,^ 27. The presence of ‘fellow strugglers’ who have experienced a similar situation makes the victims feel comfortable and less pressured to respond to any question^26^^,^ 27. The research participants wanted to participate in an interview with other women from their family (interview companions) because they felt more comfortable and emotionally supportive when sharing personal experiences of their pregnancy and birth with a stranger (the first author) in the presence of their companions.

The research participants chose their own interview companion/s whom they trusted and who was someone who had relocated with them at the times of the floods. The interview companion/s helped the interviewee to recall experiences by suggesting connecting stories that had happened to them during the floods. The group interview added richness to the data because all members contributed knowledge on the subject and provided their perspective to one story i.e. the woman’s childbirth during the floods.

The interview guidelines were carefully developed to ensure that the participants knew they did not need to answer questions which they considered embarrassing in a group setting. All the interviews were conducted in Sindhi language and audio recorded. Verbal consent was obtained from every woman, including the interview companions, prior to commencing the interview. Interviews covered topics such as women’s social and demographic characteristics, their pre-floods maternity care practices, risk perceptions and migration decisions during floods, antenatal and postnatal care, birthing experiences and social support during the floods. Ethics approval were obtained from Deakin University Human Research Ethics Committee (HREC ref: DU 2014), and the Pakistan National Bioethics Committee (NBC -161).

Interviews were transcribed verbatim then thematic analysis was conducted and key themes and codes were systematically defined. As the research project was part of a Doctoral Degree research project, data was coded by a single researcher (first author) however, themes were discussed and agreed between all the authors. NVIVO v11 was used for coding and analysis^28^. Pseudonyms have been used for research participants and the study district to maintain their confidentiality.

## Results

The main theme that emerged from the thematic analysis was poor support provided to women during different stages of floods. Women’s lack of support was categorized into three sub-themes, early warning and relocation to relief camps, living in make-shift houses in relief camps, and the third stage was when women gave birth in unhygienic conditions. Figure 1 describes the main themes and sub-themes that emerged from the data. The three sub-theme headings will form the basis of the Results section.

**Women's experiences during floods:**
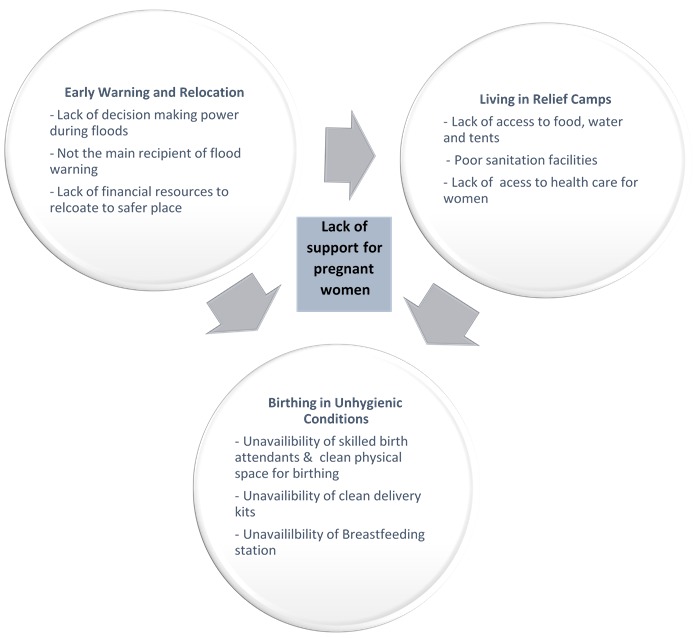

The age range of participants were from 22 to 36 years at the time of floods. The number of children women had ranged from two to ten, and six women had four children. Twelve out of fifteen women worked as agriculture or seasonal laborers and three women were housewives. None of participants had ever attended school. Table 1 shows participants’ social and demographic characteristics.

**Figure:**
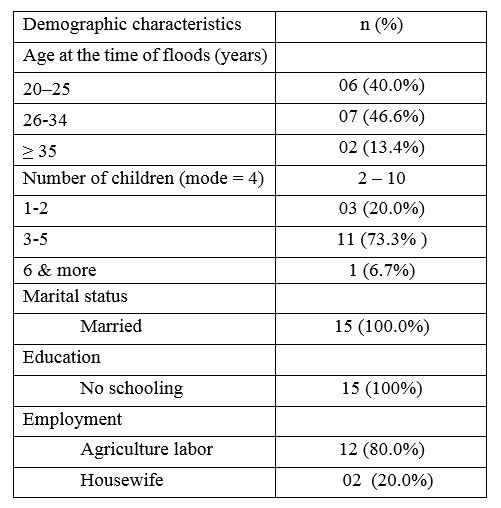
**Table 1.** Demographic characteristics of participants

*Early warning and evacuation*

The 15 women interviewed said that they received an early warning from the emergency relief officers, however their elder male kin decided that the family did not need to relocate at that time’ these families were forced to evacuate their village soon thereafter as the flood water level increased. Families from the fishing community along the Indus River said that their male kin were experienced boatmen and they considered that the flood water level would not rise to dangerous levels. However, their prediction was wrong. When the flood waters rose and descended upon their village the residents made an emergency evacuation to the relief camps.

That night was horrible. There were people screaming for help. We had to leave in a hurry as the flood water was approaching to the village. We left in a hurry, barefoot! I took my children and ran to the nearest hill where the government has set up temporary shelters (Aisha, age 35).

Families who worked as agricultural laborers delayed their relocation to safe locations because they were required to seek permission from their landowners or village leaders to leave their agricultural work. The women said that their families did not relocate when they were give an early warning because they were afraid that they may lose their employment. In all situations, whether the women relocated when they were given an early warning or not, women were never involved in the decision making about the relocation; this important decision about women’s safety and wellbeing was the responsibility of male family members, or elderly male villagers.

The district management authority made arrangements for villagers to relocate to the relief camps utilizing the existing infrastructure. They sought the help of landowners and influential political leaders who used their own transport to evacuate the villagers. However, there was not enough trailers and tractors available to take all the villagers to the relief camps so many pregnant women had to walk two to three kilometers to the relief camps, accompanied by their children; only women who could afford to rent private transport did so.

There were hundreds of people who left the house and very few trailers or tractors available for relocation. It was dark. We were not sure about what to do. Those who could afford to rent a van/car paid the money to get to the relief camps. (Shazia, age 32).

One pregnant woman mentioned that she felt scared and unsafe as she and her children rushed to the relief camp late at night. She was caught up in a swell of frightened villagers who were also trying to reach safety. She feared for her safety and that of her children as they walked through deep flood waters before she finally managed to get on a crowded bus which was going to the relief camp.

**Life in a relief camp**

All 15 women and their families relocated to the designated relief camp site ‘Sain Takkar’ in District Hussain Shah where approximately 20,000 people from nearby villages lived during the floods in 2011.

*Camp setting*

Women described their lives in the relief camps as miserable and troublesome. When they reached the relief camps they were told by the staff that there were not enough tents to give to all the residents. Families without a tent built their own temporary shelters using wood, bamboo and thatch. The women who adhered to the practice of Pardah (female seclusion) were not permitted to interact with males outside their family. These women felt very embarrassed living in an overcrowded temporary shelter with no privacy and sharing their living space with many strangers, many of whom were men.

*Access to food, water and sanitation*

Food supplies in the relief camp were irregular and they were delivered every two or three days. During the first week in the camp the residents were provided with cooked meals three times a day. However, there were only three food stalls in the camp to distribute the food to thousands of residents. Relief staff worked at each food distribution point but they were unable to organize a food distribution system for masses waiting for their food rations. Women said that it was difficult to obtain food in the first few days in the relief camp. Male family members queued for the family’s food rations while their wives, the widows and single women waited in the make shift shelters for the men to return with the food supplies.

A water tanker visited the camps three times a week. Women mentioned that they could not access the drinking water because the water points were far from their tents and on the days when the water tankers arrived in the camp there were hundreds of people in line to receive their family’s water quota. It was particularly difficult for young mothers because they could not queue at the water points for hours with their children, or leave the children in temporary shelters in the company of strangers. It is not culturally acceptable for women to wait in queues in the presence of men. Some of the women said that their family drank contaminated water from a stream near the cattle camp, knowing the health risks of drinking the water. The mismanagement associated with the distribution of food and water caused the masses of hungry people waiting to become frustrated. Many women reported incidents were the supplies were plundered by people in desperate need of food and water.

There were only three stalls where food suppliers came after 2-3 days. Imagine a mob of thousands of hungry people being catered by only a few individuals in emergency situations. The mob often got agitated. There was no proper distribution mechanism. (Aisha, age 35).

Relief camp communal toilet blocks were located a distance from the women’s temporary shelters. Postpartum women found it hard to go to the toilet during the day because the toilet block was far from their temporary shelters and it was over crowded. The temporary toilets erected in the camps were not private so it was embarrassing for women to use them during the day.

*Health facilities in relief camp*

There was one health camp set up by the DDMA to provide health services for the entire relief camp population. The staff in the health camp treated illnesses such as the common cold and fever, malaria, snake bites, skin infections, diarrhea etc. The health camp was mostly accessed by men. Women mentioned that when their children were sick, a male family member visited the health camp to get medicine on behalf of the children because there were only male doctors staffing the health camp. Women did not visit male doctors for their own health problems nor did they take their children to see a male doctor. In addition, the area near the health camp was generally crowded by men so women did not feel comfortable to go there.

We (females) did not go there (health camp) as there were too many males around that area. My husband brought medicine for my child, I couldn’t go to that camp because there were male doctors. (Shazia, age 28).

**Birthing in relief camps**

*Dysfunctional Referral system*

The camp did not provide basic obstetric facilities therefore pregnant and labouring women were provided referral slips to attend nearest rural health facilities without the transportation provision. These health facilities were located within 20 kilometers radius from health camp, however, since heavy rains worsened the district road conditions, healthcare access became an issue. Asa result, many flood affected population travelled to 50km to a civil hospital of the nearest city because the inter-city road conditions were better than the road conditions within the district. Transportation was not the part of referral system therefore people were obliged to organize their own transport. Families who had motorbikes or a bull cart helped other families to go to the hospital or at least to get to the nearest bus stop. This support was generally free of cost and in some cases the family paid for the fuel used in the motorbikes.

Aisha was 37 weeks pregnant with twins when she arrived in the relief camp. She had a poor obstetric history which included five miscarriages in four years. When she visited the health camp for antenatal care the nurse told her that the staff were not skilled to examine pregnant women or conduct deliveries and the facility was too small to provide obstetric care. Aisha was referred to the civil hospital for maternity care. Her husband borrowed money from his relatives for the transportation to the hospital, cost of medicines, and doctor’s fee.

“The female nurse said that she could not conduct a delivery because the camp was too small for birthing purpose. The doctor recommended me to go to civil hospital in district Ghulam Hyder.” (Aisha, age 35).

Women who could not afford the hospital fee and transportation cost were assisted by the traditional birth attendant (TBA), known as the Dai, in the relief camp. Zubia went into labour during the night and was referred to the district hospital. However, Zubia did not have enough money to pay for private transport to take her to the hospital so her mother, who was a Dai, assisted her to give birth in the relief camp. She was satisfied with her decision to stay in the camp and be assisted by the Dai as going to the hospital would have cost her money and caused a delay in seeking birthing care which could have placed Zubia and her baby’s life at risk.

“How could we have gone to civil hospital in another city without any transport? I didn’t have enough money to go on my own. It was night time. At the end my mother helped me to deliver the baby. She is a Dai as well. Thank God, I made a good choice and did not waste time to reach to the health facility”. (Zubia, age 31)

Another woman Haseena used TBA’s assistance for delivery because she like Zubia did not have enough money to go the hospital. She thought that if there had been trained obstetric staff working in the health camp her labour would not have been prolonged and she may have received pain relief during the labour.

*Unavailability of clean delivery kit*

A clean delivery kit is an essential obstetric supply in crisis situations especially when women give birth in unhygienic conditions, because it reduces the risk of umbilical cord and puerperal and postpartum infections [32]. Unfortunately, none of the study participant received a clean delivery kit, instead the Dai used the local birthing kit that pregnant women had made before relocating to the relief camp. Women spoke about the importance of carrying their own birthing kit with them through difficult terrains to the relief camp, as they expected to deliver their baby soon after their arrival in the camp. The birthing kit is a bag that every pregnant woman prepared when she was about 28 to 32 weeks’ gestation. The kit included baby clothes, oil for massage, soap, water bottle, blade (to cut the umbilical cord), and some extra clothing for the mother. The reason for preparing the birthing kit was because, if a laboring women was unable to access the maternity care facility or a skilled birth attendant, and the delivery was conducted by the Dai or family members, all the necessary birthing equipment was in a bag for the labouring women and her birth attendant to use.

“I made my local birthing kit after seven months. Thank God it was ready. I was expected to deliver one week after we relocated to the relief camp. At that time, I was not sure how long would we stay at relief camp. So I took all the important things just in case the Dai would have to deliver my baby. (Mariam, age 28).

**Birthing in temporary shelter**

There was no clean physical space available where women could have given birth. The health camp was too small, mostly accessed by men and always overcrowded. Most of the study participants who gave birth in relief camp did so in their own tent, or temporary shelters. Haseena described her experience of birthing in the camp as embarrassing. She gave birth outside their temporary shelter because the shelter was small and other family members such as her children and in-laws were also living there. Secondly, she gave birth in an open space without any privacy. To maintain Haseena’ privacy, seven or eight women from her Para formed a circle around Haseena and her Dai. The women used thin quilts (called rillis) or bedsheets as a barrier so that no one from outside can see Haseena giving birth.

“I gave birth outside our shelter house, as our other family members were living there as well. Seven to eight women from my para covered me and my Dai by bedsheets and thin quilts (rillis) to maintain the privacy of birthing. It was very awkward because birthing is a very private matter for any woman. (Haseena, age 32).

*Safety arrangements for lactating women and their newborns*

There was no separate, private and safe place allocated for pregnant and lactating women. Women who gave birth in the relief camps had difficulties breastfeeding their babies in the company of others, especially in front of men. Zubia considered that lactating women should have been provided with a private area to breastfeed their infants and additional support to establish breastfeeding. The shelter that Zubia’s family constructed did not have a private space to breastfeed her newborn as other family members of mixed sex also shared the living space.

After the baby was born the hardest thing was to breastfeed the baby. The shelter we made was not private and other family members were also sharing the same space. It was especially hard during the day when everyone was around, I felt really embarrassed to breastfeed in the presence of other men from our family. There must be some separate place for lactating women so that they can breastfeeding easily during the day. (Zubia, age 31).

Nishat from Syed Peerani whose family was not provided with a tent said that her family lived in the relief camp without any shelter at all. She emphasized that she was seven months pregnant and didn’t have a place to sit or rest which was very difficult. After two days in the relief camp, she decided to return to her semi damaged house in the flooded village as her health condition was deteriorating in the relief camp.

"I came back after spending two days at the camp. We were sitting without any shelter. I was 7 months pregnant. There was no place to sit or rest, I had to sleep on the floor. At least pregnant women should have access to a place where she can do a little bit of rest” (Nishat, age 31).

Ten of the fifteen women who gave birth in the relief camp left the camp within a week of giving birth. The postpartum women travelled up to 72 hours to reach their relatives’ houses because their living conditions in the camp were not appropriate for their newborn infants. Women reported cases of snakebites and malaria which heightened their concern for their newborn’s health and wellbeing, in addition to the health challenges they faced in the postpartum phase, as noted above.

## Discussion

Women’s decision making participation at various stages of disaster was non-existent. Despite the fact that women are more risk averse decision makers than men, and they are better at responding to disaster mitigation strategies, they were not the direct recipient of early warning of the floods^30^. Women’s vulnerability to natural disasters is greater in patriarchal societies where male household heads make the key decisions and female family members adhere to those decision^31^. It is noted that since the women in the study did not directly receive an early warning, they relied on the information provided to them by their household heads or other family members. Enarson also noted women’s lack of inclusion in early warning system which increases their vulnerability to poor access to basic facilities during disaster and access to relief aides at the post disaster stage^32^. Ganapati argues that the social networks in disaster situations might go against the wellbeing of women especially where women’s fate is in the hands of others^33^. We noted that unnecessary bureaucracy to obtain approval to relocate pre floods, the villagers’ low risk perception and women’s lack of participation in relocation decision making resulted in the pregnant women giving birth in unfavorable conditions.

Amaratunga emphasized that conservative societies which value physical and sexual modesty should make use of gender-sensitive camp geography, women friendly spaces, gender-sensitive medical facilities and tents, and female skilled birth attendants to reduce women’s vulnerability in disaster or conflict situations^34^. As a result of the lack of gender sensitive disaster response strategies, women faced challenges accessing basic facilities such as food, water, and shelter. Pregnant and lactating women found the camp setting was not appropriate as there was no space for them to rest and breastfeed in privacy. Given the conservative nature of Pakistani society, women friendly spaces should be part of the relief camp infrastructure which is accessible to women of all ages.

The nutritional guidelines for a malnourished population during emergency settings^14^^,^ 35 consider that there is a need to provide special access to food and nutritional supplements (iron, folic acid and vitamin A) for pregnant and lactating women, as they are at increased risk of malnutrition. Sindh Province has the highest rate of acute malnourishment in Pakistan, ranging from 23.1% in Northern Sindh and 21.2% in Southern Sindh, which is the flood affected region^36^. The study participants received regular rations supplied by the DDMA, Pakistan in the relief camps for five to seven days and they were not given additional food and nutritional supplements. Given the high rate of acute malnourishment in rural Sindh, it is immensely important to incorporate nutrition sensitive strategies at the disaster response and rehabilitation stages.

The international humanitarian recommendation for reproductive health (MISP) during natural disasters was not followed during floods^13^. In the absence of basic obstetric facilities, essential medical supplies, and a non-functional referral system, women gave birth in unhygienic and unsafe conditions with the help of family members and/or the Dai. The UNHCR recommends that in the physically hard to access areas, pregnant women and local TBAs should be provided with a clean delivery kit so that women can give birth in hygienic conditions^37^. While these kits were provided in other parts of the country by the UN agencies and the DDMA^38^^,^ 39, none of the study participant or the Dai received a clean delivery kit from any of the stakeholders. Instead Dai used the local birthing kit that pregnant women had made themselves before relocating to the relief camp. Women had no control over the choice and place of birth or their birth attendant. They made birthing decisions on the basis of the available resources not on their health condition. The absence of skilled birth attendants and a clean physical space to give birth put women and their newborn infants at significant risk of mortality.

Lactating women described their challenges of breastfeeding in the temporary shelters. The women felt exposed in the presence of men which was a matter of shame (sharam) for the women participants. This was similarly noted during the earthquake in Pakistan in 2005, many women stopped breastfeeding as they felt uncomfortable to breastfeed their babies in the presence of their distant male relatives with whom they shared shelters^40^. The lack of privacy for breastfeeding in a disaster setting will have long term implications on infants’ nutritional and growth, given the high prevalence of malnutrition and stunting in rural Pakistan.

## Recommendations

The Pakistan Government should develop and implement women inclusive disaster policies. In disaster prone regions, community health workers are required to educate pregnant women and their families about the importance of timely relocation upon receiving an early warning. A clean physical space or birthing station with essential obstetric supplies and drugs, managed by skilled birth attendants or community health workers, can significantly reduce the excess maternal morbidity and mortality in crisis situations. During disasters, every relief camp should have a birthing station situated in the health camp and should be managed by female skilled birth attendants or community health workers. The birthing station should have all essential supplies and equipment for normal births in addition to clean delivery and dignity kits or hygiene kits which include culturally appropriate clothing, sanitary pads, panties and essential toiletries^41^. These stations should be made accessible to Dais who can also utilize the basic obstetric facilities in the absence of a skilled birth attendant, and where appropriate, work alongside the skilled birth attendants. All women with an obstetric complication must be referred to a health care facility and their birth should be assisted by the EmOC trained staff in the facility. The birthing stations should be accessible 24/7 to all pregnant and birthing women and the same facility could be used as a breastfeeding station. Adequate supply of food and water along with the recommended nutritional supplements should be provided to pregnant, birthing and lactating women in disaster settings.

## Corresponding Author

Humaira Maheen: hmaheen@deakin.edu.au

## Competing Interest Statement

The authors have no financial relationships or conflicts of interest to disclose.

## Data Availability Statement

The data set contains identifying information which is unsuitable for public deposition. De-identified data are available upon request and requests may be sent to Humaira Maheen (hmaheen@deakin.edu.au)

## Appendix

**List of abbreviation**

BHU Basic Health Unit

CDC Center for Disease and Prevention

CMW Community midwife

DDMA District Disaster Management Authority

DHO District Health Officer

EmOC Emergency Obstetric Care

LHV Lady Health Visitor

LHW Lady Health Worker

MISP Minimum Initial Service Package

NDMA National Disaster Management Authority

NGO Non-Government organization

RHC Rural Health Center

SBA Skilled birth attendant

TBA Traditional birth attendant

UNFPA United Nations Population Fund

UNICEF United Nations International Children's Emergency Fund
